# Factors Associated with Colposcopy-Histopathology Confirmed Cervical Intraepithelial Neoplasia among HIV-Infected Women from Rio De Janeiro, Brazil

**DOI:** 10.1371/journal.pone.0018297

**Published:** 2011-03-30

**Authors:** Angela Cristina Vasconcelos de Andrade, Paula Mendes Luz, Luciane Velasque, Valdiléa Gonçalves Veloso, Ronaldo I. Moreira, Fabio Russomano, Janice Chicarino-Coelho, Elaine Pires, José Eduardo Levi, Beatriz Grinsztejn, Ruth Khalili Friedman

**Affiliations:** 1 Instituto de Pesquisa Clínica Evandro Chagas, Fundação Oswaldo Cruz, Rio de Janeiro, Brasil; 2 Departamento de Matemática e Estatística, Universidade Federal do Estado do Rio de Janeiro, Rio de Janeiro, Brasil; 3 Instituto Fernandes Figueira, Fundação Oswaldo Cruz, Rio de Janeiro, Brasil; 4 Laboratório de Virologia, Instituto de Medicina Tropical, Universidade de São Paulo, São Paulo, Brasil; University of Sao Paulo, Brazil

## Abstract

**Introduction:**

Despite the availability of preventive strategies (screening tests and vaccines), cervical cancer continues to impose a significant health burden in low- and medium-resourced countries. HIV-infected women are at increased risk for infection with human papillomavirus (HPV) and thus development of cervical squamous intraepithelial neoplasia (CIN).

**Methods:**

Study participants included HIV-infected women enrolling the prospective open cohort of Evandro Chagas Clinical Research Institute, Oswaldo Cruz Foundation (IPEC/FIOCRUZ). At cohort entry, women were subjected to conventional Papanicolaou test, HPV-DNA test and colposcopy; lesions suspicious for CIN were biopsied. Histopathology report was based on directed biopsy or on specimens obtained by excision of the transformation zone or cervical conization. Poisson regression modeling was used to assess factors associated with CIN2+ diagnosis.

**Results:**

The median age of the 366 HIV-infected women included in the study was 34 years (interquartile range: 28–41 years). The prevalence of CIN1, CIN2 and CIN3 were 20.0%, 3.5%, and 2.2%, respectively. One woman was found to have cervical cancer. The prevalence of CIN2+ was 6.0%. Factors associated with CIN2+ diagnosis in the multivariate model were age < years compared to ≥35 years (aPR  =  3.22 95%CI 1.23–8.39), current tobacco use (aPR  =  3.69 95%CI 1.54–8.78), nadir CD4 T-cell count <350 cells/mm3 when compared to ≥ 350 cells/mm3 (aPR  =  6.03 95%CI 1.50–24.3) and concomitant diagnosis of vulvar and/or vaginal intraepithelial lesion (aPR  =  2.68 95%CI 0.99–7.24).

**Discussion:**

Increased survival through wide-spread use of highly active antiretroviral therapy might allow for the development of cervical cancer. In Brazil, limited cytology screening and gynecological care adds further complexity to the HIV-HPV co-infection problem. Integrated HIV care and cervical cancer prevention programs are needed for the prevention of cervical cancer mortality in this group of women.

## Introduction

The HIV/AIDS pandemic continues to spread in many parts of the world. In Latin American countries, as a result of increased heterosexual transmission, there is now a growing number of HIV infected women [1]. Indeed, the impact of HIV/AIDS pandemic is most pronounced among the poorest and the youngest, with women being overrepresented in these groups [1]. Several studies have consistently identified that HIV-infected women, especially those with low CD4 T-cell counts, are at increased risk for infection with human papillomavirus (HPV) [2], [3], [4], the etiologic agent of cervical cancer and its precursor lesions. HIV-infected women also have higher rates of infection with high-risk HPV types, are frequently infected with multiple HPV types, and have a higher chance of presenting with a persistent infection [2], [3], [4]. Furthermore, HIV infection has been shown to increase a woman's risk of developing cervical squamous intraepithelial neoplasia (CIN) and invasive cervical cancer [5], [6].

Cervical cancer continues to impose a significant health burden in low- and medium-resourced countries of sub-Saharan Africa, South and South East Asia and Latin America [7], [8], [9], despite its high preventability by screening and human papillomavirus (HPV) vaccination. In Brazil, cervical cancer is the second most common form of cancer among women and the fourth cause of death by cancer [10]. In contrast to high-resource countries, middle- and low-resource countries provide little or no access to cervical cancer screening for women, regardless of HIV status [6]. Although the increased antiretroviral therapy delivery in middle- and low-resource countries has greatly decreased the burden of several opportunistic infections, the impact of the partial immune restoration induced by antiretroviral therapy on the natural history of HPV infection seems modest, at best [11]. In fact, HIV-infected women remain at a continued substantial risk for cervical neoplasia, even if they receive antiretroviral therapy [3], [6].

Studies that use cervical cytology for the diagnosis of CIN rather than histopathology are perceived as less reliable. In addition to the possibility of missing true disease (that is, of a false negative report), there is also the possibility of disease misclassification or of false positive reports when using cytology. In the ASCUS/LSIL Triage Study for Cervical Cancer (ALTS), 40% of cases classified as high-grade intraepithelial lesions with cytology that were followed with histopathology were less severe than CIN2+ during follow-up [12].

The purpose of this study was to assess the prevalence and factors associated with colposcopy-histopathology confirmed CIN in the baseline assessment of the cohort of HIV-infected women of the Evandro Chagas Clinical Research Institute, Oswaldo Cruz Foundation, located in Rio de Janeiro, Brazil.

## Methods

Ethics Statement: Written informed consent was obtained for all women. The study protocol was approved by the ethics committee of Evandro Chagas Clinical Research Institute, Oswaldo Cruz Foundation.

To study the natural history of HIV infection in women, a prospective open cohort was established at the Evandro Chagas Clinical Research Institute (IPEC), Oswaldo Cruz Foundation (FIOCRUZ), Rio de Janeiro, Brazil. Eligibility criteria include documented HIV-status and written informed consent that inquires on the willingness to participate and attend all medical appointments. Participants are included in the cohort regardless of their CD4+ cell counts or current antiretroviral status. Study visits occur at 6-months intervals when a structured questionnaire is administered inquiring on socio-demographic information, sexual and reproductive history, as well as medical history relevant to HIV/AIDS and cervical cancer (including previous sexually transmitted infections). Clinical and laboratory assessment includes a pelvic examination with specimen collection. Women with evidence of sexually transmitted infections are managed according to the Brazilian guidelines [13].

At cohort entry, women were subjected to conventional Papanicolaou (Pap) test, HPV-DNA test and colposcopy. Pap test was performed with an Ayre's wooden spatula and an endocervical brush and classified according to the Bethesda 2001 classification system. The Hybrid Capture 2 assay (HC2, Qiagen, Gaithersburg/MD/USA) with probes for high- and low-risk types was the HPV-DNA test used. Specimens were obtained with a cervical brush and stored in frozen conservative media until processing. The HC2 assay used in this study categorized HPVs into two groups: low risk (which includes HPV 6, 11, 42, 43 and 44), and high risk (which includes HPV 16, 18, 31, 33, 35, 39, 45, 51, 52, 56, 58, 59 and 68).

All women were referred to colposcopy irrespective of the Pap test results. A standardized full colposcopic evaluation including the vulvar, vaginal, and perianal regions was performed by trained gynecologists. Colposcopic results were classified according to the International Cervical Pathology and Colposcopy Federation 2002 [14].

Histopathology result was given based on directed biopsy or on specimens obtained by excision of the transformation zone or conization. These procedures were performed following the Brazilian guidelines [15] and may be summarized as follows: Large Loop Excision of the transformation zone (LLETZ) [16] was performed when directed biopsy showed CIN2+ or when the Pap test showed high-grade intraepithelial neoplasia and colposcopy was satisfactory showing major changes (in a See & Treat approach). Conization was performed when directed biopsy showed CIN2+ in women with unsatisfactory colposcopy or with a transformation zone extending beyond the first centimeter of the endocervix, or when the Pap test showed high-grade intraepithelial neoplasia and colposcopy showed major changes but was unsatisfactory. Directed biopsies were performed when minor colposcopic changes were observed but it was not possible to exclude CIN2+ and the Pap test showed LSIL, ASC or was normal. Histopathology samples from these procedures were analyzed by non-blinded pathologists from the Cervical Pathology Referral Center, located in Fernandes Figueira Institute/FIOCRUZ, who reported diagnosis by consensus. Histopathology results were classified into five categories of increasing disease severity: normal/no neoplastic abnormalities, CIN1, CIN2, CIN3, and ICC [17]. Vulvar and vaginal biopsies were classified as normal or vulvar or vaginal intraepithelial neoplasia, respectively.

The final diagnosis was based on histopathology for those who underwent invasive procedures and on colposcopy in women who had no clinical indication for undergoing invasive procedures or for whom histopathology was inconclusive. All CIN 2+/ICC diagnosis were histopathologically reported.

CD4+ T-cell counts (Becton Dickinson FACScan) were obtained from the participant's medical record. The nadir CD4+ T-cell count was defined as the lowest level of immunosuppression since HIV diagnosis up until cohort entry. The baseline CD4+ T-cell count was defined as the measured level of immunosuppression observed within 90 days of cohort entry. Women were considered to be under highly-active antiretroviral treatment (HAART) if they were under HAART for at least two months before cohort enrollment.

For the statistical analyses, the five diagnostic categories were collapsed into a binary outcome variable: negative or CIN1 (Neg/CIN1) and CIN2+. A Poisson regression analysis was used to estimate the degree of association of factors with CIN2+, using women with a Neg/CIN1 diagnosis as the reference category. For the correct estimation of the parameters, the quasi-Poisson model was used because the data showed over-dispersion when the Poisson model was applied [18], [19], [20]. A bivariate analysis was first conducted to determine which factors would enter the initial multivariate model. Factors associated with a CIN2+ diagnosis assuming a significance threshold of 20% were included in the initial multivariate model. The final model was reached by removing variables with less than 5% significance. Variables with borderline significance, but for which biological plausibility is suspected, were kept in the final model. In addition, a stratified analysis was conducted within the sub-group of women who were infected with high-risk HPV. Following the above mentioned procedures, we also estimated the factors associated with CIN2+ among women harboring high-risk HPV. All statistical procedures were carried out in R (R Software 2.9, www.r-project.org).

## Results

From May 1996 through December 2007, a total of 731 HIV-infected women enrolled into the cohort. The following women were considered ineligible for the present study: virgins (2 women), women who underwent a previous CIN treatment (3 women), and women who had undergone total hysterectomy (26 women). Eighty-nine women failed to attend their colposcopy appointment and were thus excluded. Out of the 611 women who attended their colposcopy appointment, 245 women had the exam performed more than six months from their baseline assessment and were thus excluded. Three hundred and sixty six women were included in this analysis.

The median age of the women was 34 years (interquartile range (IQR): 28–41 years), 55.1% were non-white, and 23.1% reported a maximum of four years of formal education (Table 1). Forty-three percent of the women were married or were living with a partner and 59.6% reported current sexual partners. The median age of first sexual intercourse was 17 years (IQR: 15–19 years), and the median number of lifetime sexual partners was 4 (IQR: 3–9 lifetime partners). Forty-four percent of the women reported tobacco use. The median number of pregnancies was 2 (IQR: 1–4). The median nadir CD4 count was 273 cells/mm^3^ (IQR: 133–439 cells/mm^3^) while the median baseline CD4 count was 347 cells/mm^3^ (IQR: 193–546 cells/mm^3^). Seventy-three percent of the women were HAART-naive or had used HAART for fewer than 60 days prior to cohort entry. The median time since the last Pap test was 12 months (IQR: 8–30 months). A previous history of an HPV infection was reported by 17.9% of the women. Vaginal intraepithelial neoplasia (VaIN) and/or vulvar intraepithelial (VIN) were diagnosed in 6.8% of the women.

**Table 1:** Socio-demographic, behavioral and clinical characteristics of the HIV-infected women.

Characteristic (N)	Neg/CIN1 (%)	CIN2+ (%)	Total (%)
Age (years) (N = 364)			
<35	172 (50.3)	15 (68.2)	187 (51.3)
≥35	170 (49.7)	7 (31.8)	177 (48.7)
Race/ethnicity (N = 363)			
White	153 (44.9)	10 (45.5)	163 (44.9)
Non-white	188 (55.1)	12 (54.5)	200 (55.1)
Years of formal education (N = 363)			
<5	81 (23.8)	3 (13.6)	84 (23.1)
5–8	117 (34.3)	8 (36.4)	125 (34.5)
>8	14 3(41.9)	11 (50.0)	154 (42.4)
Married/living with partner (N = 364)			
Yes	152 (44.4)	6 (27.3)	158 (43.4)
No	190 (55.6)	16 (72.7)	206 (56.6)
Age at first sexual intercourse (years) (N = 300)			
≤16	140 (41.4)	12(54.5)	185 (51.4)
>16	198 (58.6)	10 (45.5)	175 (48.6)
Current sexual partners (N = 364)			
Yes	202 (59.1)	15 (68.2)	217 (59.6)
No	140 (40.9)	7 (31.8)	147 (40.4)
Number of lifetime sexual partners (N = 361)			
0–1	35 (10.3)	3 (13.6)	38 (10.5)
2–3	96 (28.3)	2 (9.1)	98 (27.2)
4 or more	208 (61.4)	17 (77.3)	225 (62.3)
Tobacco use (N = 363)			
Yes, current tobacco use	65(19.1)	9 (40.9)	74 (20.4)
Yes, previous tobacco use	85 (24.9)	2 (9.1)	87 (24.0)
No	191 (56.0)	11 (50.0)	202 (55.6)
Number of pregnancies (N = 363)			
None	28 (8.2)	3 (8.5)	31 (8.5)
One	80 (23.5)	2 (9.1)	82 (22.6)
≥2	233 (68.3)	17(77.3)	250 (68.9)
Number of vaginal deliveries (N = 359)			
None	143 (42.4)	7 (31.8)	150 (41.8)
One	84 (24.9)	8(36.4)	92 (25.6)
≥ 2	110 (32.6)	7 (31.8)	117 (32.6)
Use of contraceptive hormones (oral/injectable) (N = 364)			
Yes	26 (7.6)	1 (4.5)	27 (7.4)
No	316 (92.4)	21 (95.5)	337 (92.6)
Nadir CD4+ T-cell count (cells/mm^3^) (N = 342)			
<350	196 (60.9)	17 (85.0)	213 (62.3)
≥350	126 (39.1)	3 (15.0)	129 (37.7)
Baseline CD4+ T-cell count (cells/mm^3^) (N = 303)			
<350	141 (49.8)	13 (65.0)	154 (50.8)
≥350	142 (50.2)	7 (35.0)	149 (49.2)
AIDS defining illness (N = 366)			
Yes	66 (19.2)	8 (36.4)	74 (20.2)
No	278 (80.8)	14 (63.6)	292 (79.8)
HAART for more than 60 days at cohort entry (N = 340)			
Yes	80 (25.2)	10 (45.5)	90 (26.5)
No	238 (74.8)	12 (54.5)	250 (73.5)
Time since last Pap test (N = 357)			
≤1 year	173 (51.6)	12 (54.5)	185 (51.8)
1–3 years	108 (32.2)	8 (36.2)	116 (32.5)
>3 years	54(16.1)	2 (9.1)	56 (15.7)
Self-reported history of HPV (N = 351)			
Yes	57 (17.3)	6 (27.3)	63 (17.9)
No/does not know	272 (82.7)	16 (72.7)	288 (82.1)
VIN and/or VAIN (N = 322)			
Yes	17 (5.6)	5 (23.8)	22 (6.8)
No	284 (94.4)	16 (76.2)	300 (93.2)

Percentages are given within the valid responses of each variable.

HAART: highly-active antiretroviral therapy, VIN: vulvar intraepithelial neoplasia, VAIN: vaginal intraepithelial neoplasia.

The final composite colposcopy-histopathology diagnoses revealed no abnormality in 74.0% (95%CI 70.0–80.0) of the women (Table 2). The prevalence of CIN1, CIN2 and CIN3 were 20.0% (95%CI 16.0–24.0), 3.5% (95%CI 1.6–5.5), and 2.2% (95%CI 0.7–3.7), respectively. One woman was found to have invasive cervical cancer. The prevalence of CIN2+ among all women was 6.0% (95%CI 4.0–9.0). High-risk HPV was detected in all women found to have CIN2/3, as well as in the patient with invasive cervical cancer. The prevalence of CIN2+ among high-risk HPV infected women was 12.8% (95%CI 7.7–18.0).

**Table 2:** Prevalence of colposcopy-histopathology confirmed cervical intraepithelial neoplasia (CIN) and invasive cervical cancer (ICC) among HIV-infected women followed at the IPEC cohort, stratified by human papillomavirus (HPV) infection.

	All women			HPV infection*			High-risk HPV		
	N	%	95% CI	N	%	95% CI	N	%	95% CI
Negative	271	74.0	[70.0–78.0]	111	60.6	[53.5–68.0]	100	58.1	[51.0–65.6]
CIN1	73	20.0	[16.0–24.0]	50	27.0	[21.0–34.0]	50	29.0	[22.2–36.0]
CIN2	13	3.5	[1.6–5.5]	13	7.0	[3.3–11.0]	13	7.6	[3.6–11.5]
CIN3	8	2.2	[0.7–3.7]	8	4.4	[1.4–7.4]	8	4.6	[1.5–7.8]
ICC	1	0.3	[0.0–0.8]	1	0.5	[0.0–1.6]	1	0.6	[0.0-1.7]
Total	366	100.0	-	183	100.0	-	172	100.0	-

*The HPV infection group includes women harboring either high-risk HPV and/or low-risk HPV.

Factors associated with CIN2+ diagnosis in the bivariate analysis were age, marital status/living with a partner, number of lifetime sexual partners, tobacco use, number of pregnancies, nadir CD4+ T-cell count, AIDS defining illness, HAART use, and intraepithelial neoplasia of the lower genital tract (VIN and/or VaIN) (p-value <0.20 for all, Table 3). Factors that remained independently associated with CIN2+ diagnosis in the multivariate model (Table 3) were age <35 years when compared to ≥35 years (aPR  =  3.22 95%CI 1.23–8.39), current tobacco use (aPR  =  3.69 95%CI 1.54–8.78), nadir CD4 T-cell count <350 cells/mm3 when compared to ≥350 cells/mm3 (aPR  =  6.03 95%CI 1.50 – 24.3) and concomitant diagnosis of VIN and/or VaIN (aPR  =  2.68 95%CI 0.99–7.24).

**Table 3:** Unadjusted and adjusted prevalence ratios (PR) for the association of factors with cervical intraepithelial neoplasia (CIN)2+ for all HIV-infected women and for those women harboring high-risk human papillomavirus (HR HPV) infection.

	All women				HR HPV infected	
	Unadjusted		Adjusted		Unadjusted	
Variable	PR	95%Cl	PR	95%Cl	PR	95%Cl
Age (years)						
<35	2.03	0.84–4.85^a^	3.22	1.23–8.39^b^	1.69	0.73–3.94^a^
≥35	1.00		1.00		1.00	
Race/ethnicity						
White	1.00				1.00	
Non-white	0.98	0.43–2.21			1.43	0.47–2.29
Years of formal education						
<5	0.50	0.14–1.73			0.53	0.16–1.79
5–8	0.89	0.36–2.17			0.92	0.36–2.18
>8	1.00				1.00	
Married/living with partner						
Yes	1.00				1.00	
No	2.04	0.82–5.09^a^			1.78	0.73–4.31^a^
Age at first sexual intercourse (years)						
≤16 years	1.64	0.72–3.71			1.77	0.80–3.90^a^
>16 years	1.00				1.00	
Current sexual partners						
Yes	1.45	0.60–3.47			1.27	0.54–3.0
No	1.00				1.00	
Number of lifetime sexual partners						
0–1	1.00				1.00	
2–3	0.26	0.04–1.47^a^			0.26	0.05–1.14
≥4	0.96	0.29–3.16			0.86	0.27–2.73
Current tobacco use						
Yes	2.70	1.18–6.17^b^	3.69	1.54–8.78^b^	3.71	1.67–8.26^b^
No	1.00		1.00		1.00	
Number of pregnancies						
None	1.00				1.00	
One	0.25	0.04–1.50^a^			0.36	0.07–1.96
≥2	0.70	0.21–2.32			1.21	0.38–3.87
Number of vaginal deliveries						
None	1.00				1.00	
≥1	1.53	0.62–3.77			1.95	0.84–4.52^a^
Use of contraceptive hormones (oral/injectable)						
Yes	0.59	0.08–4.17			0.46	0.07–3.05
No	1.00				1.00	
Nadir CD4+ T-cell count (cells/mm^3^)						
<350	3.43	1.03–11.33^b^	6.03	1.50–24.3^b^	2.47	0.77–7.84^a^
≥350	1.00		1.00		1.00	
Baseline CD4+ T-cell count (cells/mm^3^)						
<350	1.79	0.74–4.37			1.13	0.48–2.66
≥350	1.00				1.00	
AIDS defining illness						
Yes	1.97	0.79–4.88^a^			1.66	0.73–3.75
No	1.00				1.00	
HAART for more than 60 days at cohort entry						
Yes	2.31	1.02–5.22^b^			1.87	0.85–4.12^a^
No	1.00				1.00	
Time since last Pap test						
≤1 year	1.00				1.00	
1–3 years	1.06	0.44–2.54			1.11	0.48–2.60
>3 years	0.55	0.12–2.36			0.55	0.13–2.24
Self-reported history of HPV						
Yes	1.71	0.69–4.26			1.33	0.54–3.17
No/does not know	1.00				1.00	
VIN and/or VAIN						
Yes	4.26	1.60–11.29^b^	2.68	0.99–7.24	2.51	0.98–6.44^a^
No	1.00		1.00		1.00	

a p-value <0.20

b p-value <0.05

HAART: highly-active antiretroviral therapy, VIN: vulvar intraepithelial neoplasia, VAIN: vaginal intraepithelial neoplasia.

In the stratified analysis that included only women harboring high-risk HPV (Table 3), although many factors entered the initial multivariate model, the procedure of removing factors with less than 5% significance resulted in a final model with only one factor, current tobacco use. Current tobacco use was associated with a four-fold increase in the prevalence of CIN2+ (PR  =  4.41 95%CI 1.90–10.27).

## Discussion

Our study is the largest report from Latin America on CIN prevalence in HIV-infected women confirmed by a composite colposcopy-histopathology assessment. In this study, all cohort participants received a standardized diagnostic colposcopic examination. Invasive confirmatory diagnostic procedures (biopsy, LLETZ, or cervical conization) were restricted to those with colposcopic evidence of CIN, as per the Brazilian management guidelines [15]. This approach avoided unnecessary invasive diagnostic procedures while increasing the accuracy of CIN diagnosis [21]. The women followed in our cohort benefit from receiving gynecological and HIV care at the same institution which maximizes completeness of clinical, laboratory and antiretroviral use information. Also, it facilitates adherence to study procedures thus allowing for inclusion of women from different demographic and clinical profiles.

The prevalence of CIN is increased in women with HIV/AIDS [22], [23], [24]. In our study, the observed prevalence of CIN2+ was 6.0%. A limited number of studies published in the literature used colposcopy-histopathology confirmed diagnosis. A small case series in Brazil reported a CIN2+ prevalence of 12.6% (N = 87) [25] while a recent study conducted in India showed a prevalence of CIN2+ of 16.5% (N = 303) [24] among HIV-infected women using a composite colposcopy-histopathology diagnosis. Another recent study from Rwanda found a 8.8% prevalence of CIN3+ (N = 476) among HIV/HPV co-infected women [22]; an equivalent estimate from our analysis indicates a 4.9% (9/183) prevalence of CIN3+ among HPV-infected women (Table 2). Regional differences related to socio-economic factors, access to antiretroviral therapy and gynecological care, as well as differences in the profile of the women evaluated, may explain the different findings. Moreover, the different findings might also be a result of the different inclusion and exclusion criteria adopted in each study (for example, the exclusion or inclusion of women previously treated previously for CIN or women previously screened), and the lack of consensus on the systematic biopsy of colposcopic findings.

Several factors previously identified as associated with cervical cancer in the general female population were also shown to be of importance in this cohort of HIV-infected women. We found that the factors significantly and independently associated with CIN2+ diagnosis were younger age (<5 years), current tobacco use, lower nadir CD4 count (<350 cells/mm^3^) and diagnosis of intraepithelial neoplasia of the lower genital tract (VIN and/or VaIN, with borderline significance).

Women become infected with HPV soon after sexual debut and may develop CIN some years later. Accordingly, women between the ages of 30 and 40 years are expected to suffer most from CIN. Cervical cancer will generally affect women at a later age. For the HIV-infected women included in this analysis, the median age of first sexual intercourse was 17 years, and CIN2+ was most significantly associated with the age of 35 years or younger. Our finding is in accordance with other studies that have shown a higher prevalence of CIN2+ among HIV-infected women in their early/mid thirties [26], [27]. The present study also demonstrated that the prevalence of CIN2+ among the HIV-infected women who use tobacco is almost four times greater than that found among non-users. A significant association between a tobacco use and cytological abnormalities was similarly reported for the HIV-infected women of the WIHS cohort [27].

In the present study, the prevalence of CIN2+ among women with a nadir CD4 T-cell count lower than 350 cells/mm^3^ was six times greater than that observed for women with higher nadir. A similar finding was observed in the recent study from Rwanda [22]. In the Rwanda study, immunosuppression was significantly associated with CIN3+ in women harboring non-HPV16 high-risk HPV. In contrast, the recent study from India [24] did not find an association of CIN2+ prevalence with immunosuppression, though in this study immunological status was measured at study enrollment. In our study, the nadir CD4 T-cell count best reflects the level of immunodeficiency reached by the women since at cohort enrollment 27% of the women were already receiving HAART for more than 2 months. Thus, it is probable that some level of immune restoration in response to HAART had already occurred in a fraction of the women. Our result linking the level of immunodeficiency reached by the women in her lifetime with CIN2+ prevalence is consistent with the role of the immune response in controlling HPV infection and consequently CIN progression. Also, it suggests that as the CD4 T-cell count declines, vigilant follow up of the anogenital tract is warranted.

In our study, intraepithelial neoplasia of the lower genital tract was found to be significantly associated with CIN2+ in the bivariate analysis. In the multivariate model, the prevalence of CIN2+ among women diagnosed with VIN and/or VaIN was almost three times greater when compared to those who did not have such co-morbidities. Although this association only reached borderline significance, we suspect that it might be of importance given the relatively large effect size. Indeed, our sample size was small which could have impacted our power to detect a significant association of VIN and/or VaIN with CIN2+ prevalence. HIV-infected women frequently have lower genital tract intraepithelial neoplasia that is not limited to the cervix, and this has been well documented in several studies conducted in large cohorts of HIV+ women in the United States [28], [29], [30]. This finding also highlights the importance of having the vulvar, vaginal, and perianal regions of HIV-infected women evaluated during gynecologic exams and colposcopy.

Our study has limitations. The cross sectional nature of the study implies that we cannot infer causality because the temporality of the associations is unknown, but we can raise hypotheses and strengthen other evidence. The relatively small sample size can also be regarded as a limitation being a product of stringent exclusion and diagnostic criteria that, on the other hand, validates the chosen sample with respect to the absence of disease and its correct classification at baseline. Of note is the fact that the prevalence of CIN2+ among women who presented for their baseline colposcopic evaluation after 6 months of cohort entry is the same as that found among women who presented within six months, as requested in the study procedures [prevalence of CIN2+ among women who presented within 6 months of cohort entry 6.0% (95%CI 4.0–9.0) and among those who presented after 6 months of cohort entry 6.0% (95%CI 3.7–9.8)]. This finding further strengthens the validity of our exclusion criteria for the present analysis and suggests that the sample evaluated did not suffer from selection bias. Finally, we do not have data on HIV uninfected women for comparison.

In summary, our study shows a prevalence of CIN2+ of 6.0% in the baseline visit of the women with HIV/AIDS included in the IPEC/FIOCRUZ cohort. We found that the main factors associated with CIN2+ diagnosis were younger age, current tobacco use, more pronounced lifetime immunosuppression and concomitant intraepithelial neoplasia of the lower genital tract. The relative risk of invasive cervical cancer among women, as well as those living with HIV infection, varies across countries or, as is the case in Brazil, within regions of a country, on the basis of the extent to which premature death due to other causes or early detection of cancer prevents progression of precursor lesions to the invasive stage [7]. Before the introduction of antiretroviral therapy, the lack of cervical cancer screening for HIV-infected women probably had little influence on their life expectancies because of high competing mortality associated with other causes, such as tuberculosis and opportunistic infections. Currently, HIV-infected women are living longer and may thus suffer from such co-morbidity. In Brazil, universal access to HAART has been provided by the Ministry of Health since 1996 and survival is on the same order of magnitude as in high-resource countries [31]. Unfortunately, the incidence of cervical cancer among HIV-infected women has not decreased in high-resource countries since the introduction HAART [32], [33]. It is likely that cervical cancer will continue to be a significant problem especially among HIV-infected women. High-quality cervical cancer screening, including the high-coverage, has to be a public health priority. HIV/AIDS care and treatment programs thus provide an ideal platform to integrate cervical cancer prevention activities in countries that face a dual burden of both AIDS and cervical cancer.
